# Analyzing climate variations at multiple timescales can guide Zika virus response measures

**DOI:** 10.1186/s13742-016-0146-1

**Published:** 2016-10-06

**Authors:** Ángel G. Muñoz, Madeleine C. Thomson, Lisa Goddard, Sylvain Aldighieri

**Affiliations:** 1 Atmospheric and Oceanic Sciences/Geophysical Fluid Dynamics Laboratory, Princeton University, Forrestal Campus. Forrestal Road 201, Princeton, NJ USA; 2 International Research Institute for Climate and Society, Earth Institute, Columbia University, New York, NY USA; 3 Latin American Observatory for Climate Events, Centro de Modelado Científico, Universidad del Zulia, Maracaibo, Venezuela; 4 Mailman School of Public Health, Department of Environmental Health Sciences, Columbia University, New York, NY USA; 5 World Health Organization Collaborating Centre on Early Warning Systems for Malaria and other Climate Sensitive Diseases, New York, NY USA; 6 International Health Regulations/Epidemic Alert and Response, and Water Borne Diseases, Communicable Diseases and Health Analysis Department, Pan American Health Organization, Washington DC, USA

**Keywords:** Zika virus, Epidemic, Climate, Climate change, Decadal, Inter-annual, El Niño, Brazil, Drought, Vector control

## Abstract

**Background:**

The emergence of Zika virus (ZIKV) in Latin America and the Caribbean in 2014–2016 occurred during a period of severe drought and unusually high temperatures, conditions that have been associated with the 2015–2016 El Niño event, and/or climate change; however, no quantitative assessment has been made to date. Analysis of related flaviviruses transmitted by the same vectors suggests that ZIKV dynamics are sensitive to climate seasonality and longer-term variability and trends. A better understanding of the climate conditions conducive to the 2014–2016 epidemic may permit the development of climate-informed short and long-term strategies for ZIKV prevention and control.

**Results:**

Using a novel timescale-decomposition methodology, we demonstrate that the extreme climate anomalies observed in most parts of South America during the current epidemic are not caused exclusively by El Niño or climate change, but by a combination of climate signals acting at multiple timescales. In Brazil, the dry conditions present in 2013–2015 are primarily explained by year-to-year variability superimposed on decadal variability, but with little contribution of long-term trends. In contrast, the warm temperatures of 2014–2015 resulted from the compound effect of climate change, decadal and year-to-year climate variability.

**Conclusions:**

ZIKV response strategies made in Brazil during the drought concurrent with the 2015-2016 El Niño event, may require revision in light of the likely return of rainfall associated with the borderline La Niña event expected in 2016–2017. Temperatures are likely to remain warm given the importance of long term and decadal scale climate signals.

## Background

It has been postulated that the 2015–2016 El Niño-Southern Oscillation (ENSO) event or long-term climate change, contributed to the recent emergence of Zika virus (ZIKV) in Latin America and the Caribbean (LAC) [1]. While plausible, analysis of the climate–ZIKV interaction is constrained by the recent arrival of the virus in LAC, meaning there is a lack of historical time series of epidemiological data [2], and the diverse nature of prior epidemics across the globe [3]. Evidence to date suggests that ZIKV is principally transmitted by the container-breeding mosquito *Aedes aegypti* [4]. Because of its recent and rapid spread, *Ae. albopictus*, alongside other *Aedes spp*., has been identified as a minor vector, but one with significant transmission potential for the future [5]. Although ZIKV transmission depends on several factors including human behavior, it is well established that the associated vectors are sensitive to variations in environmental temperature and rainfall. Weather-based early warning systems for the related dengue virus have been suggested in different regions of the world [6–8]. Temperature is a significant driver for the development of juvenile mosquito vectors and adult feeding/egg-laying cycles, along with the length of extrinsic incubation period, and viral replication of arboviruses [8–11]. Both excess rainfall and drought have been implicated in creating breeding sites for *Aedes* vectors of ZIKV, and associated epidemics of dengue and chikungunya. Heavy rainfall may result in the development of outdoor breeding sites in a wide range of artificial containers [10, 12]; droughts may also encourage humans to change the way they store water, resulting in increases in domestic breeding sites for *Aedes spp*. [13].

The climate at any location varies from its historical average on a number of time scales, including natural year-to-year and decadal (10–30 year) variations, as well as long-term trends; the latter compatible with anthropogenic climate change signals [14]. The magnitude or persistence of climate variations may enhance or decrease epidemic potential in the region. To better understand how much of the total variance in rainfall and temperature is explained by different timescales, and how those variations connect to recent conditions associated in space and time with the emergence of ZIKV in LAC, we analyze how anomalies over time can be approximately attributed to variations in climate drivers at different timescales. This type of analysis is referred to as ‘timescale decomposition’ [14, 15]. This methodology filters the associated anomalies of a climate time-series into three components: the inter-annual, decadal, and long-term trend signals. The analysis shows how important each timescale is for explaining the entire historical climate signal observed in any particular location.

As indicated, the absence of long time-series of ZIKV transmission indices or cases prohibits a formal statistical assessment of the link between climate and ZIKV, including the epidemiological effect of the climate in 2015 on the epidemic. However, our study is based on the premise that climate is likely to be an important driver of seasonal, inter-annual and longer-term variations in ZIKV transmission, especially given that 1) temperature affects the development rates of related arboviruses and known vectors, and 2) droughts or excess rainfall influence vector breeding sites, either directly or via changes in human behavior. Our analysis therefore focuses on the particular contributions of climate signals at multiple timescales to rainfall and temperature in order to support the development of climate-informed short- and long-term strategies for ZIKV prevention and control [14].

## Data description

**Fig. 1:**
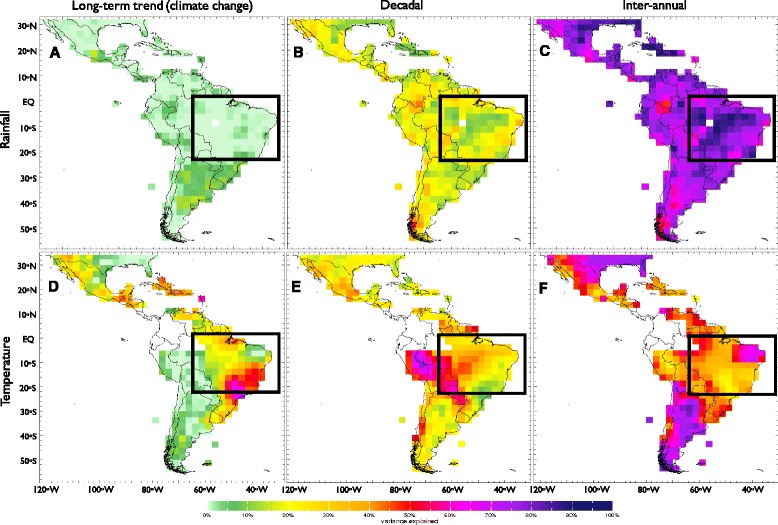
Timescale decomposition for annual precipitation (**a**–**c**) and air temperature (**d**–**f**), sketching the total explained variance for the long-term trend (**a**, **d**), decadal (**b**, **e**) and inter-annual variability (**c**, **f**) signals. Grid points in *white* indicate places where the lack of data would degrade the analysis, thus the corresponding signal has been removed by the screening process [15]. Analysis focuses in the region delimited by the *black box* (see main text)

**Fig. 2:**
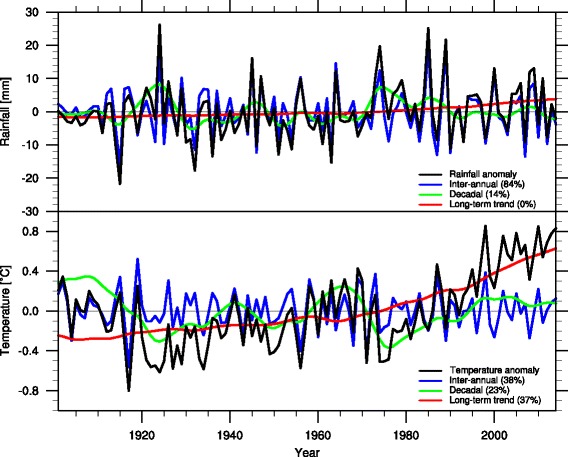
Timescale decomposition for annual anomalies in the 1901–2014 period (*black curves* represent rainfall in the *top panel*, and temperature in the *bottom panel*) averaged over the region indicated in Fig. 1 (*black box*). The anomalies correspond to the superposition of the long-term trend (*red*), the decadal signal (*green*) and the inter-annual variability signal (*blue*). Contribution of each timescale to the total explained variance is shown in *parentheses*

**Fig. 3:**
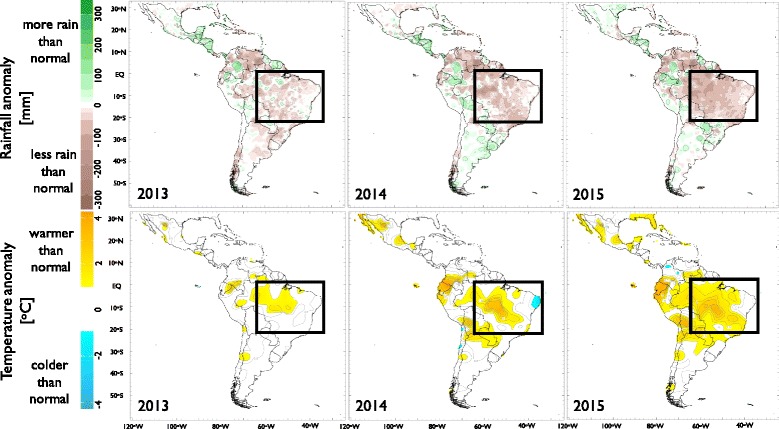
Annual rainfall (*upper row*, in mm) and temperature anomalies (*bottom row*, in °C) in Latin America and the Carribbean for 2013–2015. *White* over land indicates near-normal values. *Black box* corresponds to region with the highest number of reported Zika cases (see main text)

Since no single data set included the whole period of interest, two sources of climate data were chosen for our analysis. Timescale decomposition (Figs. 1 and 2) analysis was undertaken using the most up-to-date long-term (1901–2014) rainfall and temperature data from the University of East Anglia’s Climate Research Unit, product version 3.23 (CRUv3.23, 0.5° resolution) [16]. Recent annual temperature and rainfall anomalies (2013–2015, Fig. 3) were computed using the Climate Prediction Center’s Monthly Global Surface Air Temperature Data Set (0.5°) [17] and Rainfall Unified Data Set (0.5°) [18], respectively. Years 1979–2000 were used to compute the normal for Fig. 3.

Time series, maps and data are freely available in the International Research Institute for Climate and Society (IRI)’s Timescale Decomposition Maproom [19] and the Latin American Observatory’s Climate and Health Maproom [20, 21] for any region in the world with long enough quality-controlled records. For details, see [15].

## Results and discussion

The 20^th^ century decomposition for annual rainfall totals (Fig. 1a–c) and annual mean temperature (Fig. 1d–f) signals in LAC show sharp differences in the variability explained by each timescale. The black box overlaid onto Fig. 1 shows the area in which the highest number of reports associated with typical arbovirus vectors [22] and Zika cases [3] have been made, thus this region was selected for further analysis. On average, results for the selected region indicate that the portion of variance in rainfall associated with the climate change signal is nil (Fig. 1a), whereas that for the inter-annual component is about 60–90 % throughout the region (Fig. 1c). The decomposition also reveals that all three timescale components for surface air temperature are important (Fig. 1d–f).

The temperature long-term trend signal is particularly important in the southeastern regions of Brazil (Fig. 1d). The decadal signal is, in general, more important for temperature than for rainfall in the region, the contribution to precipitation being higher along the coast (20–30 %, Fig. 1b). For surface air temperature, however, the highest decadal component is found in the Amazon (~50 %, Fig. 1e). Inter-annual variations for surface air temperature show values over 30 % of the explained variance in most locations, with a local maximum in northeastern Brazil that explains at least 60 % of the variability (Fig. 1f). The lowest values of the explained variance at the inter-annual scale tend to correspond with the highest values of the long-term trend signal (see Fig. 1f and d).

Results are similar for the region of interest when particular seasons are considered [19, 21]: for rainfall, inter-annual and decadal scales are the most important, while for surface air temperature the three timescales share similar importance, although locally one timescale may exhibit greater importance than the others.

Complementary analysis was performed for the average climate over the boxed region of interest (Fig. 2). When summed, the specific contributions explain the observed anomalies for each particular year. These results show that a positive superposition between the rainfall inter-annual and decadal signals and all three temperature components (climate change, decadal and inter-annual) is key to understand the recent climate behavior in the region. This collection of drivers was responsible for the particularly warmer and drier than normal conditions present in the region during the last few years. The unprecedented positive temperature anomalies that started in the 1990s are consistent with the positive sign of the decadal component for that period, combined with the contributions of the long-term trend and inter-annual variability.

The spatial distribution patterns of temperature and rainfall anomalies in LAC were fairly similar in 2014 and 2015 (Fig. 3), which were, at their respective termini, the hottest years on record [23, 24]. The pattern correlations between these years are 0.81 for temperature and 0.73 for rainfall, both statistically significant (*P* < 0.05) according to a Student’s *t*-test. The year 2015 also marked the start of one of the three most intense El Niño events on record. In terms of temperature anomalies, 2013 was normal in most parts of LAC, although the warming pattern in the Amazon extending through the study region in the following years was already present. A similar claim can be made for the annual rainfall anomalies in the region under study (see black box in Fig. 3): the progressive drier than normal signal exhibited during 2014 and 2015 was already evolving in 2013. Similar anomaly patterns were present in other countries too; for example, warmer and drier than normal conditions were observed in regions of Colombia, Venezuela, Ecuador, and Puerto Rico, which have also been affected by the ZIKV epidemic.

## Conclusions

The warming observed in 2014–2015 is an outcome of positive temperature anomalies at the year-to-year and decadal timescales, superimposed on a long-term warming trend. This superposition of timescales may have helped to set the climate scenario for local ZIKV transmission via *Ae. aegypti* and other, less significant, vectors [4]. These patterns were also observed during the first half of 2016, although some rainfall anomalies have changed as the year has progressed.

As of August 2016, seasonal forecasts of sea-surface temperatures suggest that the probability of a La Niña event later this year is about 55 % [25], which is significantly higher than the corresponding climatological threshold (~35 % for the same period). La Niña events typically lead to wetter than average conditions over the northern part of Brazil and northern South America [26]. Since precipitation in this region is dominated by inter-annual variability, climate drivers at longer timescales are not likely to offset that response to La Niña. In terms of temperature, the tropics tend to be relatively cooler during La Niña events, particularly relative to El Niño. However, given the comparable magnitude of decadal variability, which currently appears to be in a warm phase, and the strength of the long-term trend, warmer than average temperatures are still the most likely outcome over the coming year, even under ENSO-neutral conditions.

The characterization of year-to-year variability and longer-term climatic trends is important for strategic activities in preparation for ZIKV outbreak in LAC and into the USA. For countries where variability and short and long-term trends are in part predictable, climate information could support the planning of prevention and control activities for different high risk areas, such as training personnel in different aspects of the outbreak early warning and response system [27].

For example, response strategies for ZIKV vector control in a warm and dry year, in which high levels of water storage provide domestic breeding sites, may need revision in a wet year when outdoor breeding sites may be more common. Current speculations about the climate drivers that may affect ZIKV transmission (see for example [1]) are based on plausible assumptions of the dynamics of the disease, but lack an in-depth understanding of the climate. However, using climate knowledge to improve health outcomes must be based on an understanding of the climate system itself and its interactions at multiple spatial and temporal scales. The timescale decomposition approach [15] used here allows a robust assessment of complex climate components to be made for any time period, season and region [19, 21]. It provides a basis for considering climate as a resource to decision-maker efforts, not only for ZIKV, but for other vector-borne diseases such as chikungunya and dengue.

## Methods

In timescale decomposition, individual gridbox values are first screened for filled data and for very dry seasons; then the time-series are detrended in order to extract slow, trend-like changes; finally, there is a filtering process, to separate high and low frequency components in the detrended data. Detrending involves regressing the local time-series on multimodel global surface air temperature data from the Twentieth Century Climate in Coupled Models [28], and low-pass filtering. Decadal components are obtained via low-pass filtering of the residual, using an order-five Butterworth filter with half-power at a period of 10 years, while the inter-annual component is computed as the difference between the residual from the detrending step and the decadal signal [15]. By construction, the method identifies the long-term trend with the anthropogenic climate change signal. For additional details, see the IRI Timescale Decomposition Maproom [19].

For the maps in Fig. 1, data were processed gridbox by gridbox, meaning that results in adjacent gridboxes are not compared or combined. For the graph of the regional time-series (Fig. 2), averaging over gridboxes was performed prior to the decomposition. Total explained variance for each component was computed for the area-averaged time-series, and not as averages of the spatial variance maps.

## Abbreviations

ENSO: El Niño-Southern Oscillation
IRI: International Research Institute for Climate and Society
LAC: Latin America and the Caribbean
ZIKV: Zika virus
